# Effects of abutment screw preload and preload simulation techniques on dental implant lifetime

**DOI:** 10.1016/j.jfscie.2022.100010

**Published:** 2022-05-20

**Authors:** Megha Satpathy, Rose M. Jose, Yuanyuan Duan, Jason A. Griggs

**Affiliations:** a Biomedical Materials Science, University of Mississippi Medical Center, Jackson, MS.

**Keywords:** Implants, finite element analysis, fatigue, failure, friction, mechanical testing

## Abstract

**Background.:**

This study aimed to investigate how the predicted implant fatigue lifetime is affected by the loss of connector screw preload and the finite element analysis method used to simulate preload.

**Methods.:**

A dental implant assembly (DI1, Biomet-3i external hex; Zimmer Biomet) was scanned using microcomputed tomography and measured using Mimics software (Materialise) and an optical microscope. Digital replicas were constructed using SolidWorks software (Dassault Systèmes). The material properties were assigned in Abaqus (Dassault Systèmes). An external load was applied at 30° off-axial loading. Eight levels of connector screw preload (range, 0–32 Ncm) were simulated for DI1. This assembly and an additional model (DI2) having a longer and narrower screw were compared regarding their fatigue limits (using fe-safe software [Dassault Systèmes]) for 2 preloading methods: (1) adding preload torque or (2) adding bolt axial tension.

**Results.:**

The maximum von Mises stresses of DI1 (on the connector screw threads) with and without preload were 439.90 MPa and 587.90 MPa. The predicted fatigue limit was the same for preloads from 100% through 80% of the manufacturer^’^s recommendation and dropped precipitously between 80% and 70% preload. Adding a preload torque on the screw resulted in a more uniform stress distribution on the screw compared with bolt axial tension, especially for DI2, which had a longer and narrower screw than DI1.

**Conclusions.:**

A substantial loss of preload can be accommodated without compromising the fatigue resistance of this dental implant. Computer models should be constructed using torque instead of a bolt axial tension.

## Introduction

Finite element analysis (FEA) is widely used for numerically solving differential equations used in mathematical modeling and engineering. It subdivides a larger system into smaller elements by space discretization using a meshing tool. The formulation of a static equilibrium results in a system of algebraic equations for each element. The equations used for modeling these finite elements are then assembled into a larger system of equations that help model the entire problem. Matrix algebra is then used to approximate the solution.^1^ FEA is a powerful tool that is widely used in various disciplines, including aeronautic, biomedical, and automotive industries, and has significantly improved both the standard of engineering designs and the methodology of the design process.^2^ It can perform simulated structural, fluid, thermal, and electromagnetic analyses. For structural analysis, it allows detailed visualization of where structures bend or twist on the application of impact or load and indicates the distribution of stresses and displacements. Using FEA, entire structures can be constructed, refined, and optimized before the prototype is manufactured.

**Figure 1:**
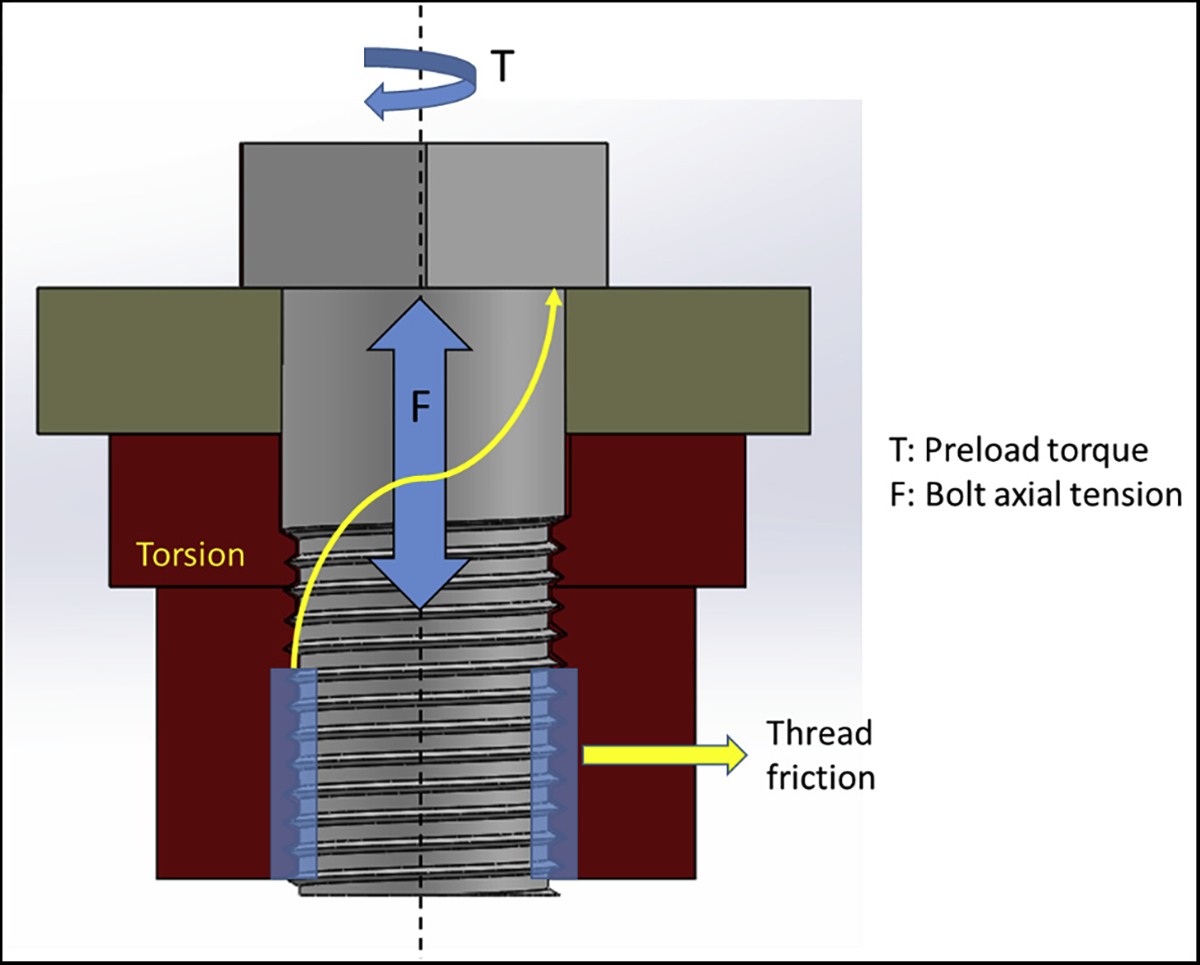
Preload application on a screw to fix 2 components. Application of the torque develops a tensile force on the surface of the screw.

FEA has been widely used in dentistry to study the stress patterns in various types of prostheses, including dental implants and fixed dental prostheses.^3–6^ The loading condition plays a vital role in the performance of dental implants.^7,8^ A multicomponent dental implant assembly consists of an implant fixture, an abutment, and a connector screw. Generally, a torque is applied to the connector screw using a torque wrench to fix the abutment to the implant fixture adequately. This tightening develops an axial tension on the surface of the screw that is in contact with the adjacent material. This is referred to as preload, as shown in Figure 1. When the screw is tightened against a material, it allows the screw to distribute the force through the material, so the screw itself only holds a portion of the load. This means that the screw can hold a significantly higher load when the correct amount of tension is applied.^9^ The magnitude of preload holds major importance in the success of dental implants. Most 2-piece dental implants fail because of screw loosening.^10–19^ This may be because of the micromotion between the implant fixture and the abutment that can lead to reduced preload on the connector screw.

In contrast, excessive preload can cause damage to the screw threads and lead to plastic deformation.^20–22^ Hence, applying the manufacturer recommended preload to the screw is essential. Adding the recommended preload may help a screw encounter a more favorable combination of stress amplitude and mean stress, resulting in increased fatigue lifetime.

This study investigated the effect of preload on the predicted fatigue lifetime of a commercially available dental implant. It was hypothesized that adding a preload will significantly improve the fatigue lifetime of the dental implant assembly. There are 2 ways in which a preload can be applied to a screw in a finite element model. The first method is to apply the preload torque directly to the screw, simulating the tightening of the screw using a torque wrench. The second method is to calculate the bolt axial tension (in N) from the recommended torque using a mathematical formula and then apply the tension on the screw. It was hypothesized that adding the torque would provide a more uniform stress distribution on the screw than the bolt axial tension method.

## Methods

The physical specimens of a dental implant assembly (DI1) including a narrow-diameter dental implant (Biomet-3i external hex; Zimmer Biomet), an abutment (GingiHue; Zimmer Biomet), and a connector screw (Gold-Tite Square screw; Zimmer Biomet) were scanned using microcomputed tomography (micro-CT) (Skyscan 1172; Micro Photonics). The scanning parameters used were as follows: an accelerating voltage of 100 kV, current of 100 μA, exposure time of 1,264 ms per frame, aluminum and copper filter, and rotation step at 0.7°. The x-ray beam was projected in a direction parallel to the long axis of the implant fixtures. The image pixel size was 34.6 μm. The x-ray projections were reconstructed to form a 3-dimensional (3D) model, saved as a stack of bitmap-type 3D files using NRecon software (Micro Photonics). Beam hardening correction of 49% and ring artifact correction of 4 were used for the reconstruction. The 3D models were generated in Mimics (Materialise) through image segmentation from the stacked image data obtained from micro-CT. Mimics organizes all the imported tomograph image slices and displays objects in 3 cross-sectional views (axial, coronal, sagittal planes). Based on the gray scale values, the objects were modified with the help of segmentation tools. The implant dimensions were measured using Mimics and an optical microscope (Keyence). The length and maximum diameter of the implant fixture were 15.12 mm and 3.40 mm, respectively.

The digital replicas of the physical specimens of DI1 were constructed using computer-aided design software (SolidWorks) (see supplemental figure [available online at the end of this article]). A hemispherical loading cap was constructed in SolidWorks to simulate a dental crown, and an 11 mm moment arm was modeled from the central point of the loading cap to the simulated bone level (as required by ISO 14801).^23^ A cylindrical bone model with 2 layers (cortical and cancellous bone) was also constructed in SolidWorks based on the dimensions of a simulated bone specimen holder that better mimics the clinical case.^24^ The bone crest was placed at 3 mm below the implant nominal bone level to simulate the worst-case scenario of crestal bone loss, based on the requirement of ISO 14801.^23^ An additional model (DI2) with a longer and narrower screw than the connector screw of DI1 was also constructed in SolidWorks. The length and a body diameter of the connector screw of DI1 were 7.34 mm and 2.10 mm, respectively. The length and a body diameter of DI2 were 8.81 mm and 1.67 mm, respectively.

FEA was performed in Abaqus (Dassault Systèmes). Table 1 shows the material properties assigned to the different components of DI1 and DI2. The material properties were assumed to be homogenous, isotropic, and linearly elastic. Boundary conditions were applied to the external nodes of the bone holder to constrain its motion on application of load. Two types of analyses were performed:

1. Analysis 1. This analysis was performed to evaluate the effect of preload on the implant fatigue limit. A preload torque of 32 Ncm was applied to the screw of DI1. An external load was applied to the loading cap at an angle of 30° from the implant axis to simulate the bite force (ISO 14801).^23^ The analysis was performed, and the .odb files were imported to fe-safe (Dassault Systèmes) software to estimate the fatigue limit using Brown-Miller criteria with Morrow mean stress correction for the lifetime calculation, as detailed in a previous study.^3^ Constant and cyclic loading (stress ratio, 0.1) were used for the preload and external load. First, a low load value was used to predict the fatigue limit, which resulted in an infinite lifetime. Then, a high load value was used resulting in a finite lifetime. The load interval was then cut into one-half, and the lifetime was predicted for the intermediate load value. This process was repeated until a threshold value was obtained, and the lifetime of the implant ceased to be infinite. After processing, the results were written to an output .odb file, which was displayed in the postprocessor of Abaqus. This entire process was repeated for the same DI1 model for connector screw preloads of 95%, 90%, 80%, 70%, 50%, 30%, and 0% of the manufacturer^’^s recommended torque (32 Ncm), and its fatigue limit was estimated in fe-safe.
2. Analysis 2. This analysis was performed to estimate the correct method for preload simulation. For this analysis, both DI1 and DI2 models were taken into consideration. Two methods were followed for preload simulation in each model. The first method was to directly add a preload torque of 32 Ncm on the connector screw and analyze the von Mises stress distribution. A reference point was created at the center of the connector screw head, and a coupling interaction was assigned between the reference point and the screw. The torque was then applied to this reference point that propagated the torque to the entire screw. The second method was to convert the preload torque (in Ncm) to bolt axial tension (in N) and then apply this tension on the connector screw in which the screw head meets the screw shank. The following equation was used to convert torque into bolt axial tension^25^:
(1) $$\text{T}=\frac{F}{2}\left[\frac{\text{p}}{\pi }+\frac{\mu_{t}d_{2}}{\cos\beta }+D_{e}\mu_{n}\right]$$
  where
  T = preload torque (Nmm)
  *F* = preload (N)
  p = screw thread pitch (mm)
  π = the ratio of the circumference of any circle to the diameter of that circle
  μ_*t*_ = coefficient of friction between screw and implant
  *d*_*2*_ = pitch diameter of screw thread (mm)
  β= half angle of screw thread (rad)
  *D*_*e*_ = (do + di)/2 (mm), where di is the inner bearing and do is the outer bearing diameter of the nut face (mm)
  μ_*n*_ = coefficient of friction between screw and abutment

Table 2 shows the values of the parameters in this equation and the calculated bolt axial tension values for DI1 and DI2. FEA was performed without applying external load to investigate the stress distribution generated only because of the effect of preload application.

## Results

### Analysis 1

**Figure 2:**
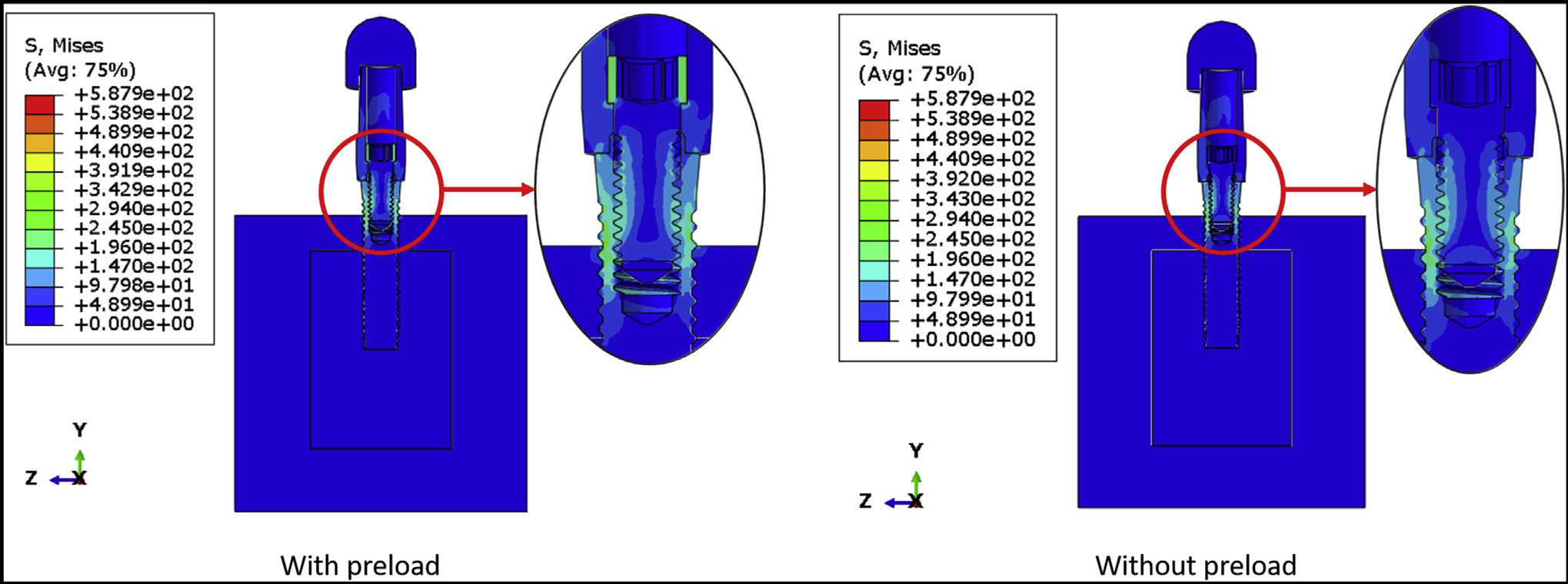
The von Mises stress distribution on the DI1 model with and without preload application.

**Figure 3:**
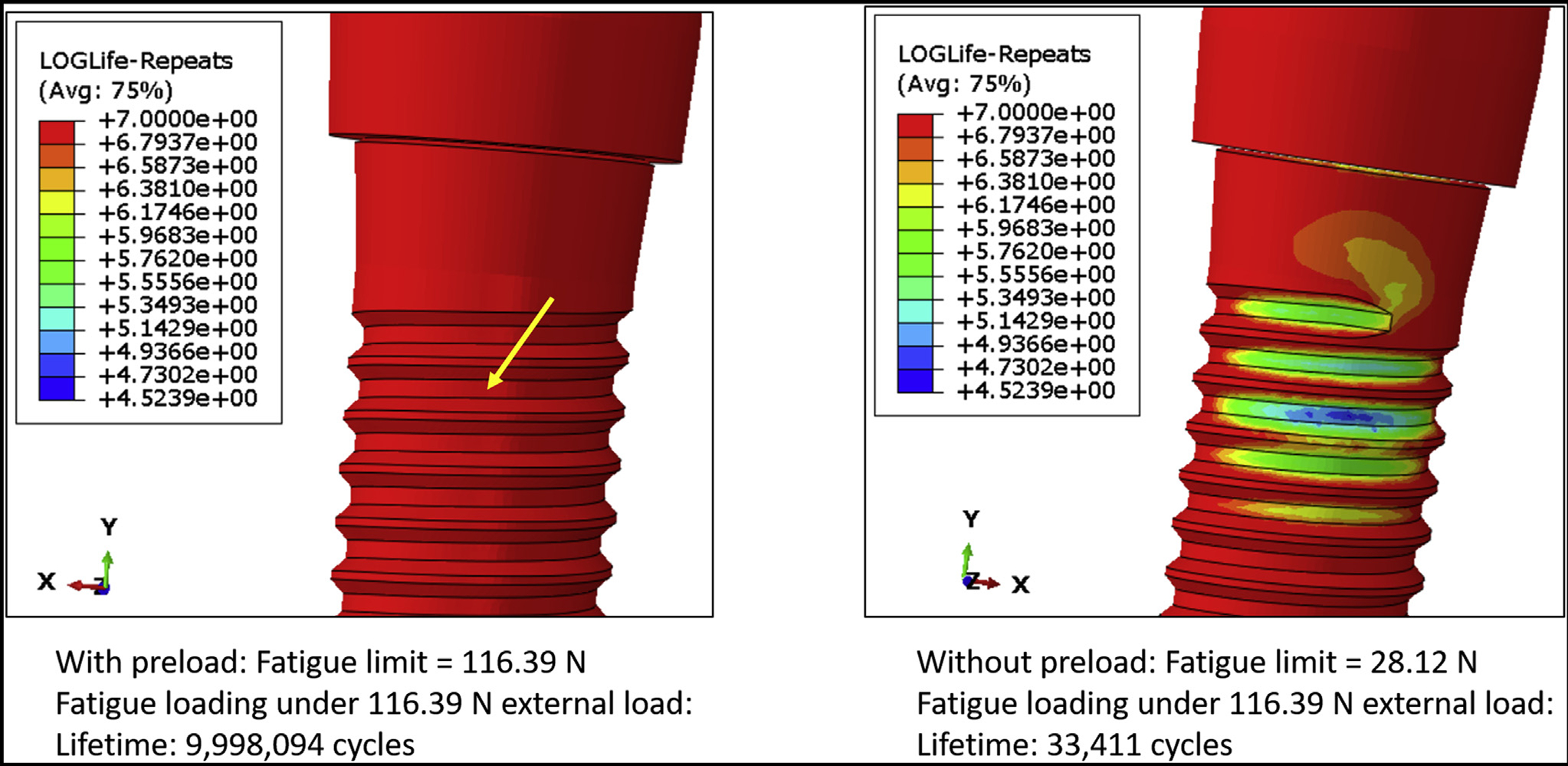
The locations of fatigue failure on the DI1 model, as predicted by fe-safe (Dassault Systémes) software. The fatigue limit of the model with preload applied was substantially higher than the fatigue limit of the model without preload. Under an external load of 116.39 N, the lifetime of the model with preload (9 × 10^6^ cycles) was substantially longer than the lifetime of the model without preload (33,411 cycles).

Figure 2 shows the von Mises stress distribution of the DI1 model with and without preload. The maximum von Mises stresses were 439.90 MPa and 587.90 MPa for the model with and without preload, respectively. In both cases, high stresses were observed on the superficial threads of the implant fixture, and the highest stresses were observed in connector threads. However, grade 4 commercially pure titanium (implant fixture) is less resistant to fatigue fracture than stainless steel (connector screw), so the fracture is predicted in the implant fixture. Figure 3 shows the fatigue failure location in the DI1 model, as predicted by fe-safe software. The fatigue limit of the model with preload applied was 116.39 N, and it was substantially higher than the fatigue limit of the model without preload (28.12 N). The lifetime of the implant (with preload applied to connector screw) under 100 N external load was infinite. Hence, to make a comparative analysis of the lifetimes and estimate the location of fatigue failure, an external load of 116.39 N was applied to both models. Under this external load, the lifetime of the model with preload was about 9 × 10^6^ cycles, which was substantially longer than the lifetime of the model without preload (33,411 cycles). The fatigue failure location in both cases was at the implant superficial threads. Table 3 shows the predicted fatigue limits for intermediate values of connector screw preload. The predicted fatigue limit did not decrease after decreasing the preload to 80% of the manufacturer^’^s recommended torque, but it dropped sharply between 80% and 70%.

### Analysis 2

**Figure 4:**
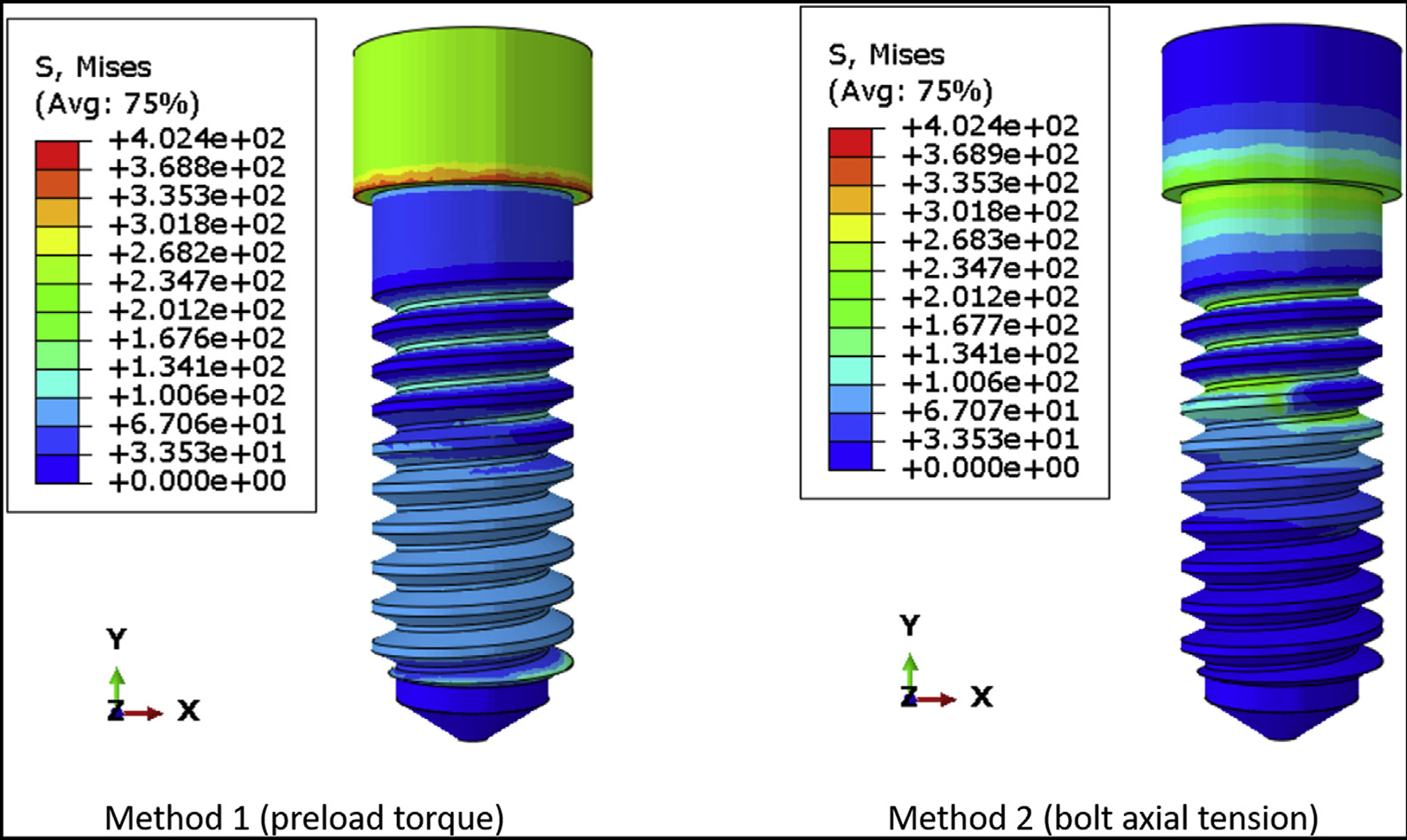
The von Mises stress distribution on the connector screw of DI1 when preload is simulated in torque and bolt axial tension.

**Figure 5:**
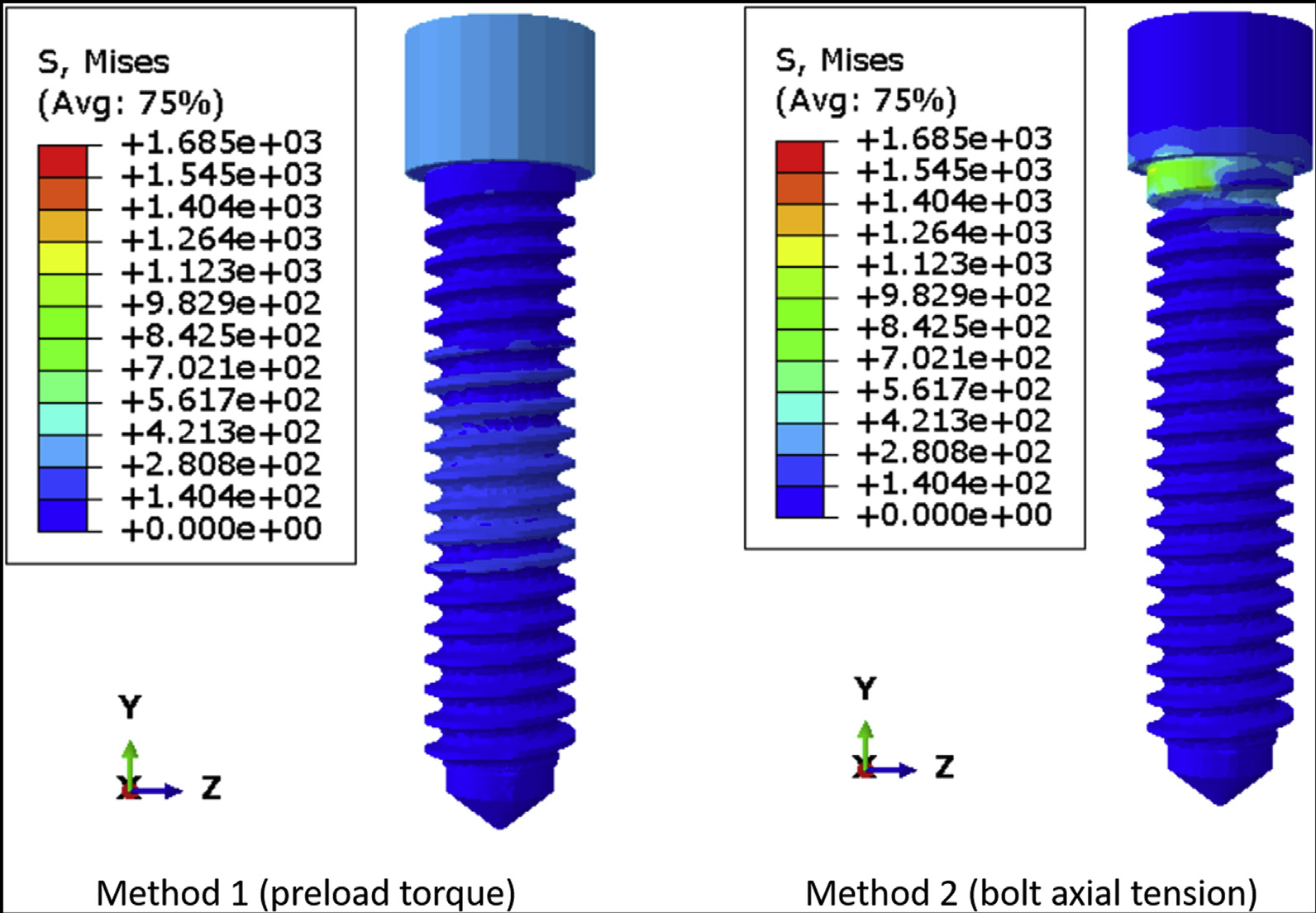
The von Mises stress distribution on the connector screw of DI2 when preload is simulated in torque and bolt axial tension.

Figure 4 shows the von Mises stress distribution on DI1 when the preload is simulated as torque and bolt axial tension. Preload torque resulted in a relatively even distribution of stress along the body of the screw, whereas bolt axial tension caused higher stress concentration around the region of the force application. This is more evident in DI2, which has a longer, narrower screw (Figure 5) than DI1. The average axial tension in the connector screw was similar regardless of whether the preload was simulated using a torque or a bolt tension. For DI1, the axial tension was 274.28 N and 290.37 N for torque application and bolt tension, respectively. For DI2, the axial tension was 352.64 N and 365.35 N for torque application and bolt tension. In DI1, the coronal one-half of the connector screw had lower stress than the apical one-half of the screw (Figure 4), and a similar pattern was observed in DI2, in which the coronal and apical one-thirds of the connector screw had lower stress than the middle one-third (Figure 5). In both DI1 and DI2, the more highly stressed region was near the bone level because of flexure at the same height at which the implant fixture predicted the lowest lifetime.

## Discussion

This study establishes the importance of applying adequate preload to the connector screw while predicting fatigue lifetime. From analysis 1, it is evident that adding a preload improved the fatigue lifetime of the dental implant significantly. This is reasonable because applying an appropriate amount of preload ensures the adequate tightening of the screw between the abutment and implant fixture. This will reduce the chances of early micromotion between the components, otherwise leading to a loss of preload resulting in early failures at the connector region from fatigue crack growth under flexure. Although performing simulations or in vitro testing on a commercially available implant, it is important to simulate a preload on the screw. However, the decrease in predicted fatigue limit with decreasing preload was not gradual. The decrease did not begin until the preload was below 80% of the recommended value, and then there was a sudden decrease in the predicted fatigue limit between 80% and 70% of the recommended preload. This provides a useful threshold for future researchers to define preliminary failures when conducting in vitro testing on physical specimens.

In several FEA studies on 2 piece dental implants, it is not clear whether the preload was applied on the connector screw of the implant assemblies, which raises a concern regarding the accuracy of the results reported from those simulations.^26–28^ Some studies have simulated preload in the form of bolt axial tension while performing FEA on dental implants.^29,30^ In this study, it was observed that simulating preload in the form of bolt axial tension can cause excessive stress concentration around the region of force application, and it may not lead to an effective distribution of stress along the body of the screw. This was more evident in the screw having a greater length and shorter diameter. Hence, it is recommended to simulate preload in the form of torque rather than bolt axial tension while performing fatigue lifetime analysis in FEA to ensure accurate simulation of preload that will improve the fatigue lifetime of the dental implant. Because the application of bolt tension at the location in which the connector screw head meets the screw shank resulted in stress concentration, another plane of tension application was tried (halfway down the screw). This method did not provide a better result, so the details of the results were not included in this report. Each method has its type of artifact. For the torque application method, Figures 4 and 5 show the head of the connector screw in a state of moderately high stress. This type of stress state is proper for some types of implant abutments that have a faceted socket to lock the screw head during torque application, but in this case, the screw head should be largely stress-free, and the stress at the screw head is an artifact of our method of torque application. It is applied at the top center at which the torque driver would engage the screw in a real specimen. One of the questions tested in this study was whether to apply the preload as a torque instead of bolt tension. The torque method of applying preload resulted in stress concentration far away from the location of likely failure, so we viewed this as less objectionable than the stress concentration on the screw threads caused by bolt tension. Saint-Venant principle indicates that the artifact from torque loading did not affect the fatigue limit or fatigue lifetime results.

The simulations in this study were performed according to the ISO 14801 standard; hence, the bone crest was placed at 3 mm below the implant nominal bone level to simulate crestal bone loss.^23^ However, FEA studies have their limitations. The materials were assumed to be homogenous, isotropic, and linearly elastic to simplify the FEA study and reduce the calculation time, but that may not reflect the clinical cases. A lack of corrosion was also assumed in the models while performing stress and fatigue lifetime analysis. The cylindrical holder was constructed on the basis of a novel bone specimen holder that attempts to mimic the clinical case,^24^ but this may not necessarily reflect the anatomic structure of the jaw bone. However, in this study, the focus was to analyze the stresses and fatigue lifetime of the implant assembly; hence, the architecture of the bone did not project a major relevance, and the cylindrical model was used to reduce the complexity of the analysis. FEA studies are based on the approximation of the results and should always be validated by performing mechanical tests. In a previous study, the results of fe-safe software were validated when the load amplitude predicted by FEA to result in failure at 1 million cycle of a narrow-diameter dental implant was within the 95% CI of the load amplitude obtained from the step-stress accelerated lifetime testing on the physical specimens of the dental implant.^3^ This study was the beginning of a larger project in which we will perform validation once an optimal implant design has been predicted.

## Conclusions

In this study, we investigated the effect of preload on implant fatigue lifetime and compared torque with bolt axial tension to determine the appropriate method to simulate preload. Within the limitations of this study, we concluded that adding a preload improved the fatigue lifetime of a dental implant assembly significantly. However, a substantial loss of preload can be accommodated without compromising the fatigue resistance of the dental implant studied. Adding a torque instead of bolt axial tension to simulate preload can generate a more accurate stress distribution on the connector screw. Hence, both the initial hypotheses were accepted.

## Supplementary Material

1

## Figures and Tables

**Table 1:** Material properties assigned to each component in Abaqus (Dassault Systèmes).

Component	Material	Young Modulus (GPa)	Poisson Ratio

Loading cap	316L SS^26^	180	0.30
Abutment	Titanium-6aluminum-4vanadium^27^	110	0.31
Connector screw	316L SS^26^	180	0.30
Implant body	Grade 4 commercially pure titanium^28^	103	0.34
Cancellous bone	Cancellous bone^29^	14	0.30
Cortical bone	Cortical bone^30^	20	0.30

**Table 2:** The values of the parameters of equation (1) for DI1 and DI2 models, and their calculated bolt axial tension (in N).

Abutment Screw Parameters	DI1	DI2

Preload torque (N mm)	320	320
Screw thread pitch (mm)	0.42	0.34
Coefficient of friction between screw and fixture	0.42	0.42
Pitch diameter of screw thread (mm)	1.89	1.46
Half angle of screw thread (rad)	0.47	0.32
Inner bearing diameter of the nut face (mm)	2.15	1.77
Outer bearing diameter of the nut face (mm)	2.57	2.22
Coefficient of friction between screw and abutment	0.50	0.50
Preload (N)	290.37	365.35

**Table 3:** The predicted fatigue limit of DI1 resulting from several different levels of connector screw preload.

Connector Screw Preload	%	Fatigue Limit (N)

32.0 Ncm	100	116
30.4 Ncm	95	117
28.8 Ncm	90	117
25.6 Ncm	80	119
22.4 Ncm	70	38
16.0 Ncm	50	37
9.6 Ncm	30	36
0.0 Ncm	0	28

## Why Is This Important?

Dental implant researchers often use finite element modeling software to predict the fatigue lifetimes of hypothetical implants. Such studies often fail to simulate the preload on the screw that connects the implant abutment to the fixture. In addition, there are multiple strategies for including the preload in the computer model. The authors determined the importance of including a preload in all simulations and the best method for doing so. For a simulated clinical bite force, the inclusion of preload increased the predicted fatigue lifetime by 6 orders of magnitude. Using a simple bolt tension to simulate preload resulted in an unrealistic stress concentration, so applying torque and appropriate coefficient of friction is the preferred modeling method. This study will allow future dental implant researchers to construct more accurate computer models. In addition, this study showed that fatigue resistance is not affected by decreased preload until the preload falls below 80% of the manufacturer^’^s recommended value. The fatigue limit then decreased precipitously between 80% and 70% preload. This defines a useful threshold for preliminary failure in future studies.
