# Supplementary material for: Effects of abutment screw preload and preload simulation techniques on dental implant lifetime
Source: JADA Found Sci. Author manuscript; Available in PMC 2023 Jan 25. (PMC9873498; doi:10.1016/j.jfscie.2022.100010)
Supplement: Supplementary file 1 [file NIHMS1862629-supplement-1.docx]

Supplemental Figure. Digital replicas of the DI1 implant assembly components constructed in SolidWorks. (The images are not of the same size scale).
